# Supplementary material for: The Clinical Implications of Inappropriate Therapy in Community-Onset Urinary Tract Infections and the Development of a Bayesian Hierarchical Weighted-Incidence Syndromic Combination Antibiogram
Source: Antibiotics (Basel). 2025 Feb 12;14(2):187. doi: 10.3390/antibiotics14020187 (PMC11851549; doi:10.3390/antibiotics14020187)
Supplement: Supplementary file 1 [file antibiotics-14-00187-s001.zip › antibiotics-3449631-supplementary.pdf]

SUPPLEMENTARY MATERIALS

Supplementary Tables

Table S1. Extrapolations and assumptions applied in cases of missing breakpoints or MIC values for antimicrobial agents on clinical microbiology reports.

Table S2. Frequencies of initial empirical antibiotic regimens in patients with UTI requiring hospitalization.

Table S3. Univariate analysis of factors associated with mortality and extended hospital stays.

Table S4. Multivariate logistic regression analyses regarding in-hospital mortality

Table S5. Multivariate logistic regression analyses regarding extended hospital stay.

Table S6. Weighted incidence syndromic combination antibiogram for urinary tract infection. Median estimated coverages (expressed as percentages) and 95% HDIs for monotherapies and combined regimens in the WISCA model.

Table S7. $\hat{R}$ Index values of WISCA model parameters in patients with community-onset urinary tract infections.

Table S8. Univariate analysis of non-susceptibility to fluoroquinolones in patients with community-onset urinary tract infections.

Table S9. Univariate analysis of non-susceptibility to third-generation cephalosporins in patients with community-onset urinary tract infections.

Table S10. Weighted incidence syndromic combination antibiogram in patients with community-onset urinary tract infections across sub-groups.

Table S11. $\hat{R}$ Index Values of WISCA model parameters in patients with community-onset urinary tract infections across sub-groups.

Supplementary Figures

Figure S1. Monte Carlo Markov Chains (MCMC) traceplots of the CoUTI WISCA model parameters.

Figure S2. Density plots of the posterior distributions of the CoUTI WISCA model parameters.

Figure S3. Autocorrelation plots for distal lags ($h \geq 2$) in posterior samples of the CoUTI WISCA model parameters.

Supplementary Tables

Table S1. Extrapolations and assumptions applied in cases of missing breakpoints or MIC values for antimicrobial agents on clinical microbiology reports.

Organism and antibiotic	Assumptions made
Pseudomonas aeruginosa	
Tigecycline	Not covered
Piperacillin-tazobactam	Covered if covered by ceftazidime unless tested and resistant
Ertapenem	Not covered
Ceftriaxone	Not covered
Nitrofurantoin	Not covered

Staphylococcus aureus	
Meropenem	If resistant to methicillin, then not covered by any beta-lactam
Piperacillin-tazobactam	If resistant to methicillin, then not covered by any beta-lactam
Cephalosporins	If resistant to methicillin, then not covered by any beta-lactam
Enterobacterales	
Ceftazidime	Covered if covered by ampicillin and ceftriaxone
Piperacillin-tazobactam	Covered if covered by ampicillin-sulbactam
	Covered if covered by ampicillin
	Covered if covered by piperacillin
Meropenem	Covered if covered by ampicillin-sulbactam
	Covered if covered by ampicillin
	Covered if covered by piperacillin unless tested and resistant
	Covered if covered by piperacillin-tazobactam unless tested and resistant
Ertapenem	Covered if covered by ampicillin-sulbactam
	Covered if covered by ampicillin
	Covered if covered by piperacillin unless tested and resistant
	Covered if covered by piperacillin-tazobactam unless tested and resistant
Ampicillin	*K. pneumoniae* not covered
	Enterobacter cloacae not covered
Gram-negative organisms	
Linezolid	Not covered
Vancomycin	Not covered
Enterococcus species	
Ertapenem	Not covered
Meropenem	Resistant for *E. faecium*; *E. faecalis* resistant if resistant to ampicillin.
Fosfomycin	*E. faecium* not covered
Acinetobacter baumannii	
Nitrofurantoin	Not covered
Ertapenem	Not covered
Proteus mirabilis	
Nitrofurantoin	Not covered
Candida species	
Candida glabrata	Not covered by antibacterial antibiotics
Candida tropicalis	Not covered by antibacterial antibiotics

Table S2. Frequencies of initial empirical antibiotic regimens in patients with UTI requiring hospitalization.

Initial Empirical Scheme	Number	Percentage
Ertapenem	87	35.95
Ceftriaxone	59	24.38
Meropenem	24	9.92
Levofloxacin	21	8.68
Ciprofloxacin	8	3.31
Piperacillin/Tazobactam	8	3.31
Fosfomycin	7	2.89
Amikacin	6	2.48
Meropenem + Linezolid	6	2.48
Nitrofurantoin	4	1.65
Ceftriaxone + Ciprofloxacin	2	0.83
Cephalothin	1	0.41
Clindamycin	1	0.41
Ceftriaxone + Metronidazole	1	0.41
Cefotaxime	1	0.41
Ertapenem + Amikacin	1	0.41
Ertapenem + Linezolid	1	0.41
Fosfomycin + Amikacin	1	0.41
Meropenem + Amikacin	1	0.41
Meropenem + Vancomycin	1	0.41
Trimethoprim/Sulfamethoxazole	1	0.41

Variable	Obs.	In-hospital mortality				Obs.	Extended hospital stays**			
		Total (n = 242)	Deaths (n = 34)	Survivors (n = 208)	Significance*		Total (n = 242)	Yes (n = 29)	No (n = 213)	Significance*
Age -mean (IQR)	242	53 (40.0-63.0)	60 (55.5-67.0)	51 (37.8-63.0)	**	242	53 (40.0-63.0)	56 (46.0-72.0)	53 (40.0-63.0)	NS
Women -n (%)	242	165 (68.2)	24 (70.6)	141 (67.8)	NS	242	165 (68.2)	17 (58.62)	148 (69.48)	NS
Men -n (%)	242	77 (31.8)	10 (29.4)	67 (32.2)		242	77 (32.8)	12 (41.38)	65 (30.52)	
Complicated UTI - n (%)	242	235 (97.1)	34 (100.0)	201 (96.6)	NS	242	235 (97.10)	29 (100.0)	206 (96.70)	NS
Permanent urinary catheter - n (%)	242	28 (11.6)	4 (11.8)	24 (11.5)	NS	242	28 (11.6)	1 (3.4)	27 (12.7)	NS
Recurrent UTI - n (%)	242	93 (38.4)	12 (35.3)	81 (38.9)	NS	242	93 (38.4)	11 (37.9)	82 (38.5)	NS
Pyelonephritis - n (%)	242	235 (97.1)	34 (100.0)	201 (96.6)	NS	242	235 (97.1)	29 (100.0)	206 (96.70)	NS
Initial empirical inappropriate treatment - n (%)	239	72 (30.1)	16 (47.1)	56 (27.3)	*	242	72 (30.1%)	9 (31.0)	63 (30.0)	NS
Days to correction of empirical treatment - median (IQR)	55	6 (2.0-4.0)	3 (2.0-3.0)	3 (2.0-4.0)	NS	55	3 (2.0-4.0)	3 (3.0-5.0)	3 (2.0-4.0)	NS
Inappropriate final treatment in UTI event – n (%)	239	20 (8.4)	7 (20.6)	13 (6.3)	*	242	20 (8.4)	5 (17.2)	15 (7.1)	NS
Previous antibiotic treatment within 90 days median (IQR)	25	1 (6.0-15.0)	5 (5.0-9.5)	7 (6.3-15.0)	NS	25	7 (6.0-15.0)	5.5 (5.0-8.0)	7 (7.0-15.0)	NS
Previous hospitalization within 90 days - n (%)	242	108 (44.6)	14 (41.2)	94 (45.2)	NS	242	108 (44.6)	13 (44.8)	95 (44.6)	NS
**Severity of Illness**										
Hypotension - n (%)	242	56 (23.1)	31 (91.2)	25 (12.0)	**	242	56 (23.1)	8 (27.6)	48 (22.5)	NS
Need for vasopressors - n (%)	242	45 (18.6)	30 (88.2)	15 (7.2)	**	242	45 (18.6)	7 (24.1)	38 (17.8)	NS
SOFA Score - median (IQR)	242	2 (0.0-6.0)	9 (7.3-10.8)	2 (0.0-4.0)	**	213	2 (0.0-6.0)	6 (2.0-8.0)	2 (0.0-6.0)	**
**Comorbidities**										
Charlson comorbidity index - median (IQR)	242	3 (1.0-5.0)	6 (5.0-7.0)	3 (1.0-4.0)	**	242	3 (1.0-5.0)	3 (2.0-5.0)	3 (1.0-5.0)	NS
Diabetes mellitus - n (%)	242	150 (62)	30 (88.2)	120 (57.7)	**	242	150 (62.0)	20 (69)	130 (61.0)	NS
Hypertension - n (%)	242	82 (33.9)	19 (55.9)	63 (30.3)	**	242	82 (33.9)	11 (37.9)	71 (33.3)	NS
Cardiovascular disease - n (%)	242	48 (19.8)	15 (44.1)	33 (15.9)	**	242	48 (19.8)	8 (27.6)	40 (18.8)	NS
Acute kidney injury - n (%)	242	105 (43.4)	28 (82.4)	77 (37)	**	242	105 (43.4)	22 (75.9)	83 (39.0)	**
Chronic kidney disease - n (%)	242	68 (28.1)	23 (67.6)	45 (21.6)	**	242	68 (28.1)	10 (34.5)	58 (27.2)	NS
Chronic liver disease - n (%)	242	4 (1.7)	0 (0.0)	4 (1.9)	NS	242	4 (1.7)	0 (0.0)	4 (1.9)	NS
Pregnancy - n (%)	242	9 (3.7)	0 (0.0)	9 (4.3)	NS	242	9 (3.7)	1 (3.4)	8 (3.8)	NS
Immunosuppression - n (%)	242	14 (5.8)	2 (5.9)	12 (5.8)	NS	242	14 (5.8)	2 (6.9)	12 (5.6)	NS
Cancer - n (%)	242	15 (6.2)	5 (14.7)	10 (4.8)	*	242	15 (6.2)	2 (6.9)	13 (6.1)	NS
Central nervous system Neurological disease- n (%)	242	28 (11.6)	4 (11.8)	24 (11.5)	NS	242	28 (11.6)	1 (3.4)	27 (12.7)	NS
Peripheral neuropathy- n (%)	242	26 (10.7)	7 (20.6)	19 (9.1)	NS	242	26 (10.7)	6 (20.7)	20 (9.4)	NS
**Outcome**										
Urinary tract infection related complications	242	68 (28.1)	31 (91.2)	37 (17.8)	**	242	68 (28.1)	13 (44.8)	55 (25.8)	NS
Local complication of UTI - n (%)	242	29 (12.0)	12 (35.3)	17 (8.2)	**	242	29 (12%)	5 (17.2%)	24 (11.3%)	NS
System complication of UTI n (%)	242	48 (19.8)	30 (88.2)	18 (8.7)	**	242	48 (19.8)	8 (27.6)	40 (18.8)	NS
Hospital stay - median (IQR)	242	6.5 (4.0-10.0)	5 (3.0-10.0)	7 (4.0-10.0)	NS	-	-	-	-	-

Prolonged hospital stay - n (%)	242	29 (12.0)	5 (14.7)	24 (11.5)	NS	-	-	-	-	-

Table S3. Univariate analysis of factors associated with mortality and extended hospital stays.

* Significance levels: **: $p < 0.01$; *: $p < 0.05$; NS: Not significant. **Defined as a stay exceeding the 90th percentile of our patient population (15 days).

Table S4. Multivariate logistic regression analyses regarding in-hospital mortality

Model	Variable	OR	CI Lower	CI Upper	Significance*
Model A	Inappropriate initial antibiotic treatment	1.439	0.416	5.057	NS
	SOFA Score	1.735	1.348	2.396	**
	Charlson comorbidity index	1.214	0.869	1.696	NS
	Urinary tract infection related complications	7.207	1.674	41.478	*
	Age	1.013	0.957	1.07	NS
	Chronic kidney disease	3.025	0.852	11.994	NS
	Cardiovascular disease	1.091	0.282	4.003	NS
Model B	Inappropriate final antibiotic treatment	10.506	1.311	135.265	*
	SOFA Score	1.719	1.333	2.368	**
	Charlson comorbidity index	1.192	0.859	1.671	NS
	Urinary tract infection related complications	10.758	2.218	84.270	**
	Age	1.004	0.949	1.063	NS
	Chronic kidney disease	3.205	0.877	13.402	NS
	Cardiovascular disease	1.053	0.256	4.157	NS

Model A: Hosmer–Lemeshow p value= 0.8546; Pseudo R^2 = 0.708
Model B: Hosmer–Lemeshow p value= 0.9754; Pseudo R^2 = 0.728

* Significance levels: **: p < 0.01; *: p < 0.05; NS: Not significant.

Table S5. Multivariate logistic regression analyses regarding extended hospital stay.

Model	Variable	OR	CI Lower	CI Upper	Significance*
Model A	Inappropriate initial antibiotic treatment	1.723	0.752	3.91	NS
	SOFA Score	1.017	0.891	1.155	NS
	Acute kidney injury	3.887	1.445	11.455	**
	Age	1.012	0.984	1.040	NS
Model B	Inappropriate final antibiotic treatment	2.675	0.757	8.392	NS
	SOFA Score	1.013	0.886	1.151	NS
	Acute kidney injury	4.387	1.640	13.013	**
	Age	1.010	0.982	1.039	NS

Model A: Hosmer–Lemeshow p value= 0.7507; Pseudo R^2 = 0.129
Model B: Hosmer–Lemeshow p value= 0.1156; Pseudo R^2 = 0.135

* Significance levels: **: p < 0.01; *: p < 0.05; NS: Not significant.

Table S6. Weighted incidence syndromic combination antibiogram for urinary tract infection. Median estimated coverages (expressed as percentages) and 95% HDIs for monotherapies and combined regimens in the WISCA model.

Antibiotic regimen	WISCA with Bayesian hierarchical design	

	Median coverage (%)	Lower 95% HDI (%)	Upper 95% HDI (%)	WISCA without Bayesian design
Ciprofloxacin	9.047	3.279	22.351	21.488
Ceftriaxone	18.127	7.032	39.016	37.603
Ceftriaxone + Vancomycin	18.678	7.332	39.688	38.430
Ceftazidime	18.678	7.272	39.882	36.364
Ceftriaxone + Linezolid	18.976	7.444	40.120	38.843
Trimethoprim/Sulfamethoxazole	19.471	7.489	41.737	21.901
Cefepime	20.224	7.993	42.084	36.364
Ceftazidima + Vancomicina	20.433	8.062	42.582	40.083
Ceftazidima + Linezolid	20.693	8.197	42.891	40.496
Cefepime + Vancomicina	20.861	8.333	43.174	37.190
Cefepime + Linezolid	21.263	8.450	44.031	37.603
Nitrofurantoína	52.822	26.899	77.317	39.256
Amikacina	64.506	37.676	84.296	67.355
Ceftriaxona + Amikacina	70.254	43.912	87.484	78.099
Ceftazidima + Amikacina	70.313	43.962	87.595	74.793
Cefepime + Amikacina	70.747	44.343	87.889	75.620
Piperacilina/Tazobactam	77.205	50.268	91.946	34.711
Piperacilina/Tazobactam + Vancomicina	80.934	55.631	93.675	35.537
Piperacilina/Tazobactam + Linezolid	82.737	58.243	94.401	35.950
Ertapenem	84.997	64.420	94.692	89.256
Ertapenem + Vancomicina	87.546	69.101	95.848	90.496
Fosfomicina	87.772	66.510	96.462	38.430
Ertapenem + Linezolid	88.525	70.656	96.172	90.909
Piperacilina/Tazobactam + Amikacina	90.252	72.907	96.976	74.380
Meropenem	90.889	75.389	97.108	91.736
Meropenem + Vancomicina	92.643	78.804	97.767	92.562
Meropenem + Linezolid	93.429	80.726	98.072	92.975
Ertapenem + Amikacina	95.065	84.177	98.633	92.562
Meropenem + Amikacina	95.137	84.235	98.678	93.388

Regimens ordered from lowest to highest coverage. HDIs, Highest Density Intervals.

Table S7. $\hat{R}$ Index values of WISCA model parameters in patients with community-onset urinary tract infections.

Parameter	R_hat
b_Intercept	1.00075019
sd_pathogen__Intercept	1.00010579
sd_regimen__Intercept	1.00260851
Intercept	1.00075019
r_pathogen[Acinetobacter.baumannii,Intercept]	1.00012858
r_pathogen[Candida.glabrata,Intercept]	1.00020529
r_pathogen[Candida.parapsilosis,Intercept]	0.9999965
r_pathogen[Candida.tropicalis,Intercept]	1.00030839
r_pathogen[Citrobacter.freundii,Intercept]	1.0004041
r_pathogen[Entero.faecalis,Intercept]	1.00067048
r_pathogen[Entero.faecium,Intercept]	1.00032826
r_pathogen[Enterobacter.cloacae,Intercept]	1.00040903
r_pathogen[Escherichia.coli,Intercept]	1.00109376

r_pathogen[Klebsiella.pneumoniae,Intercept]	1.00102414
r_pathogen[Morganella.morganii,Intercept]	1.00029104
r_pathogen[Proteus.mirabilis,Intercept]	1.00111232
r_pathogen[Pseudomonas.aeruginosa,Intercept]	1.00087325
r_pathogen[Staphylococcus.aureus,Intercept]	1.00086064
r_pathogen[Staphylococcus.saprophyticus,Intercept]	1.00000928
r_regimen[AN,Intercept]	1.00051009
r_regimen[CAZ,Intercept]	1.00049495
r_regimen[CIP,Intercept]	1.00051683
r_regimen[combo_CAZ_AN,Intercept]	1.00044753
r_regimen[combo_CAZ_LNZ,Intercept]	1.00045784
r_regimen[combo_CAZ_VA,Intercept]	1.00059041
r_regimen[combo_CRO_AN,Intercept]	1.0004059
r_regimen[combo_CRO_LNZ,Intercept]	1.0003826
r_regimen[combo_CRO_VA,Intercept]	1.00068729
r_regimen[combo_ETP_AN,Intercept]	1.00045389
r_regimen[combo_ETP_LNZ,Intercept]	1.00051362
r_regimen[combo_ETP_VA,Intercept]	1.00024364
r_regimen[combo_FEP_AN,Intercept]	1.00053615
r_regimen[combo_FEP_LNZ,Intercept]	1.0005618
r_regimen[combo_FEP_VA,Intercept]	1.00033009
r_regimen[combo_MEM_AN,Intercept]	1.00014851
r_regimen[combo_MEM_LNZ,Intercept]	1.0000808
r_regimen[combo_MEM_VA,Intercept]	1.00020702
r_regimen[combo_TZP_AN,Intercept]	1.00042299
r_regimen[combo_TZP_LNZ,Intercept]	1.00009245
r_regimen[combo_TZP_VA,Intercept]	1.00024357
r_regimen[CRO,Intercept]	1.00051504
r_regimen[ETP,Intercept]	1.00028543
r_regimen[FEP,Intercept]	1.00055382
r_regimen[FOS,Intercept]	1.00014427
r_regimen[MEM,Intercept]	1.0001331
r_regimen[NIT,Intercept]	1.00057327
r_regimen[SXT,Intercept]	1.00040615
r_regimen[TZP,Intercept]	1.00008339
lprior	1.00019526
lp__	1.00063072

Table S8. Univariate analysis of non-susceptibility to fluoroquinolones in patients with community-onset urinary tract infections.

Variable	Obs.	Total (n = 229)	Present (n = 177)	Absent (n = 52)	Significance*
Age - mean (IQR)	229	54 (41.0-63.0)	54 (42.0-64.0)	52.5 (40.8-63.0)	NS
Women, - n (%)	154	154 (67.2)	122 (68.9)	32 (61.54)	NS
Men - n (%)	75	75 (32.8)	55 (31.1)	20 (38.46)	
Complicated UTI - n (%)	229	223 (97.4)	172 (97.2)	51 (98.1)	NS
Pyelonephritis - n (%)	229	223 (97.4)	172 (97.2)	51 (98.1)	NS
Permanent urinary catheter - n (%)	229	28 (12.2)	19 (10.7)	9 (17.3)	NS
Recurrent UTI - n (%)	229	92 (40.2)	73 (41.2)	19 (36.5)	NS

Previous antibiotic treatment within 90 days - mean (IQR)	22	7 (6.0-15.0)	7 (6.0-15.0)	3 (2.0-16.5)	NS
Inappropriate initial antibiotic treatment - n (%)	227	70 (30.8)	57 (32.6)	13 (25.0)	NS
Days to correction of empirical treatment - median (IQR)	55	3 (2.0-4.0)	3 (2.0-4.0)	4 (2.5-4.0)	NS
Inappropriate final antibiotic treatment in UTI event – n (%)	227	18 (7.9)	16 (9.1)	2 (3.8)	NS
Previous hospitalization within 90 days - n (%)	229	104 (45.4)	74 (41.8)	30 (57.7)	NS
**Severity of Illness**					
Hypotension - n (%)	229	54 (23.6)	44 (24.9)	10 (19.2)	NS
Need for Vasopressors - n (%)	229	43 (18.8)	39 (22.0)	4 (7.7)	*
SOFA Score - median (IQR)	229	2 (0.0-6.0)	2 (0.0-6.0)	2 (0.0-3.0)	NS
Charlson comorbidity index - median (IQR)	229	3 (2.0-5.0)	3 (2.0-5.0)	3 (1-4.25)	NS
**Comorbidities**	229	48 (21.0)	34 (19.2)	14 (26.9)	NS
Diabetes mellitus - n (%)	229	143 (62.4)	117 (66.1)	26 (50.0)	NS
Hypertension - n (%)	229	78 (34.1)	63 (35.6)	15 (28.8)	NS
Cardiovascular disease - n (%)	229	44 (19.2)	41 (23.2)	3 (5.8)	**
Acute kidney injury - n (%)	229	103 (45.0)	80 (45.2)	23 (44.2)	NS
Chronic kidney disease - n (%)	229	64 (27.9)	55 (31.1)	9 (17.3)	NS
Chronic liver disease - n (%)	229	3 (1.3)	3 (1.7)	0 (0.0)	NS
Pregnancy - n (%)	229	8 (3.5)	6 (3.4)	2 (3.8)	NS
Immunosuppression - n (%)	229	14 (6.1)	9 (5.1)	5 (9.6)	NS
Cancer - n (%)	229	13 (5.7)	9 (5.1)	4 (7.7)	NS
Central Nervous System Neurological Disease- n (%)	229	28 (12.2)	19 (10.7)	9 (17.3)	NS
Peripheral Neuropathy- n (%)	229	24 (10.5)	21 (11.9)	3 (5.8)	NS
**Outcome**					
Urinary Tract Infection related complications	229	66 (28.8)	60 (33.9)	6 (11.5)	**
Local Complication of UTI - n (%)	229	29 (12.7)	27 (15.3)	2 (3.8)	NS
Systemic Complication of UTI n (%)	229	46 (20.1)	41 (23.2)	5 (9.6)	NS
Hospital stay - median (IQR)	229	7 (4.0-10.0)	7 (4.0-10.0)	6.5 (4.0-10.0)	NS
Prolonged hospital stay - n (%)	229	28 (12.2)	22 (12.4)	6 (11.5)	NS

*Significance levels: **: p < 0.01; *: p < 0.05; NS: Not significant.

Table S9. Univariate analysis of non-susceptibility to third-generation cephalosporins in patients with community-onset urinary tract infections.

Variable	Obs.	Total (n = 242)	Present (n = 151)	Absent (n = 91)	Significance*
Age - mean (IQR)	242	53 (40-63)	53 (40-65.5)	53 (40.5-63.0)	NS
Women, - n (%)	242	165 (68.2)	98 (64.9)	67 (73.6)	NS
Men - n (%)	242	77 (31.8)	53 (35,1)	24 (26.4)	
Complicated UTI - n (%)	242	235 (97.1)	145 (96.0)	90 (98.0)	NS
Pyelonephritis - n (%)	242	235 (97.1)	145 (96.0)	90 (98.9)	NS
Permanent urinary catheter - n (%)	242	28 (11.6)	18 (11.9)	10 (11.0)	NS
Recurrent UTI - n (%)	242	93 (38.4)	66 (43.7)	27 (29.7)	*
Inappropriate initial antibiotic treatment - n (%)	239	72 (30.1)	60 (40.3)	12 (13.3)	-
Days to correction of empirical treatment - median (IQR)	55	3 (2.0-4.0)	3 (2.5-4.0)	2 (2.0-3.0)	NS
Inappropriate final antibiotic treatment in UTI event – n (%)	239	20 (8.4)	19 (12.8)	1 (1.1)	**
Previous hospitalization within 90 days - n (%)	242	108 (44.6)	72 (47.7)	36 (39.6)	NS
Previous antibiotic treatment within 90 days	25	7 (6-15)	7 (6-15)	5(2.5-9)	NS
**Severity of Illness**					
Hypotension - n (%)	242	56 (23.1)	43 (28.5)	13 (14.3)	*
Need for Vasopressors - n (%)	242	45 (18.6)	34 (22.5)	11 (12.1)	NS

SOFA Score - median (IQR)	242	2 (0.0-6.0)	3 (0-6.0)	2 (0-4.5.0)	NS
Charlson comorbidity index - median (IQR)	242	3 (1.0-5.0)	3 (2-5)	3 (1.0-5.0)	NS
**Comorbidities**	242	190 (78.5)	119 (78.8)	71 (78.0)	NS
Diabetes mellitus - n (%)	242	150 (62.0)	96 (63.6)	54 (59.3)	NS
Hypertension - n (%)	242	82 (33.9)	51 (33.8)	31 (34.1)	NS
Cardiovascular disease - n (%)	242	48 (19.8)	33 (21.9)	15 (16.5)	NS
Acute kidney injury - n (%)	242	105 (43.4)	71 (47.0)	34 (37.4)	NS
Chronic kidney disease - n (%)	242	68 (28.1)	44 (29.1)	24 (26.4)	NS
Chronic liver disease - n (%)	242	4 (1.7)	2 (1.3)	2 (2.2)	NS
Pregnancy - n (%)	242	9 (3.7)	4 (2.6)	5 (5.5)	NS
Immunosuppression - n (%)	242	14 (5.8)	11 (7.2)	3 (3.3)	NS
Cancer - n (%)	242	15 (6.2)	11 (7.3)	4 (4.4)	NS
Central Nervous System Neurological Disease- n (%)	242	28 (11.6)	18 (11.9)	10 (11)	NS
Peripheral Neuropathy- n (%)	242	26 (10.7)	14 (9.3)	12 (13.2)	NS
**Outcome**					
Urinary Tract Infection related complications	242	68 (28.1)	53 (35.1)	15 (16.5)	**
Local Complication of UTI - n (%)	242	29 (12)	20 (13.2)	9 (9.9)	NS
Systemic Complication of UTI n (%)	242	48 (19.8)	37 (24.5)	11 (12.1)	**0.0293**
Hospital stay - median (IQR)	242	6.5 (4.0-10.0)	7 (3.0-11.0)	6 (4.0-8.0)	NS
Hospital stay > 15 days - n (%)	242	29 (12)	23 (15.2)	6 (6.6)	NS

* Significance levels: **: $p < 0.01$; *: $p < 0.05$; NS: Not significant.

Table S10. Weighted incidence syndromic combination antibiogram in patients with community-onset urinary tract infections across sub-groups.

	18-30 years			30-65 years			>65 years		
regimen	mean_median_pct	mean_hdi_lower_pct	mean_hdi_upper_pct	mean_median_pct	mean_hdi_lower_pct	mean_hdi_upper_pct	mean_median_pct	mean_hdi_lower_pct	mean_hdi_upper_pct
AN	83.12	66.31	95.91	67.57	62.16	72.74	60.56	51.07	69.59
CAZ	48.66	27.89	69.96	46.77	36.70	55.62	32.13	19.13	44.70
CAZ+AN	80.57	63.95	93.37	65.97	60.30	71.21	56.06	46.35	64.96
CAZ+LNZ	47.17	26.64	68.42	48.53	38.94	57.03	32.04	19.39	44.38
CAZ+VA	47.10	26.72	68.71	48.54	38.86	56.92	30.64	17.88	43.07
CIP	20.12	5.73	40.26	35.15	23.77	46.30	27.31	14.83	40.21
CRO	48.75	28.28	70.62	46.41	36.33	55.27	30.98	18.28	43.37
CRO+AN	80.52	63.79	93.44	68.35	63.20	73.40	57.13	47.62	65.84
CRO+LNZ	47.26	26.74	68.23	47.00	37.07	55.73	32.02	19.44	44.39
CRO+VA	47.26	26.98	68.93	47.02	37.16	55.80	30.68	18.08	43.18
ETP	96.92	89.69	99.95	73.75	69.22	79.09	65.77	57.14	74.35
ETP+AN	96.94	89.71	99.92	77.59	72.57	84.13	67.29	58.74	75.98
ETP+LNZ	96.92	89.85	99.93	74.70	70.18	80.30	68.89	60.45	77.81
ETP+VA	96.92	89.77	99.91	74.69	70.19	80.35	67.27	58.85	75.92
FEP	51.89	29.01	74.78	48.23	38.50	56.84	32.85	19.82	45.63
FEP+AN	71.03	52.23	87.74	67.31	61.88	72.34	57.14	47.62	66.01
FEP+LNZ	40.08	20.85	62.24	47.04	37.05	55.76	30.64	18.16	43.23
FEP+VA	40.02	20.42	61.85	47.02	37.00	55.71	29.26	16.54	41.85
FOS	94.88	82.41	99.93	73.58	68.04	79.93	68.53	58.10	78.74
MEM	96.91	89.77	99.94	75.73	71.11	81.62	70.58	62.10	79.50
MEM+AN	96.92	89.76	99.90	78.34	73.14	85.10	68.89	60.34	77.56
MEM+LNZ	96.90	89.77	99.93	76.93	72.12	83.21	70.68	62.17	79.73
MEM+VA	96.90	89.80	99.92	76.90	72.14	83.26	68.90	60.42	77.64
NIT	79.56	59.70	94.86	64.25	57.21	70.66	54.66	43.40	64.85
SXT	45.56	22.05	70.63	46.07	34.97	55.78	41.88	28.02	54.42
TZP	93.07	74.44	99.90	69.87	63.90	75.77	65.70	54.70	75.89
TZP+AN	74.31	55.86	89.61	65.97	60.32	71.17	57.11	47.67	65.90
TZP+LNZ	32.81	14.43	54.22	44.66	34.39	54.01	35.95	23.56	47.93
TZP+VA	32.82	14.49	54.64	44.67	34.40	53.95	34.70	22.35	46.67

Group	Non-recurrent UTI			Recurrent UTI			Antibiotic treatment < 90 days absent			Antibiotic treatment < 90 days present		
regimen	median_pct_reg	hdi_lower_pct_reg	hdi_upper_pct_reg	median_pct_reg	hdi_lower_pct_reg	hdi_upper_pct_reg	median_pct_reg	hdi_lower_pct_reg	hdi_upper_pct_reg	median_pct_reg	hdi_lower_pct_reg	hdi_upper_pct_reg
AN	63.84	57.86	69.58	67.60	58.86	75.98	63.82	58.60	68.57	63.40	54.76	71.66
CAZ	45.88	36.08	54.76	37.10	25.51	48.14	45.19	35.45	54.16	32.43	22.07	42.42
CAZ+AN	63.05	57.11	68.73	62.35	53.29	70.76	62.09	56.54	67.04	60.90	52.22	69.11
CAZ+LNZ	46.66	37.12	55.33	38.91	27.57	49.68	46.07	36.53	54.87	34.39	24.26	44.24
CAZ+VA	46.30	36.64	54.99	38.95	27.73	49.76	46.10	36.54	54.90	33.61	23.49	43.56
CIP	32.94	21.59	44.54	29.41	18.00	41.20	27.81	17.55	38.80	31.04	20.63	41.33
CRO	45.44	35.62	54.39	36.89	25.62	47.89	45.46	35.78	54.32	30.46	20.23	40.79
CRO+AN	64.56	58.78	70.18	65.11	56.47	73.38	64.42	59.62	68.94	61.60	53.11	69.94
CRO+LNZ	45.88	36.10	54.65	37.42	25.98	48.23	45.71	36.05	54.49	31.98	21.67	42.02
CRO+VA	45.52	35.76	54.40	37.44	26.01	48.34	45.68	35.99	54.49	31.17	20.86	41.25
ETP	71.24	65.82	77.14	75.30	67.75	83.47	70.17	66.33	75.89	70.70	62.73	78.27

ETP+AN	74.19	68.67	80.68	79.39	71.70	87.99	72.19	67.68	79.50	75.80	68.35	83.09
ETP+LNZ	73.36	67.89	79.61	76.25	68.69	84.40	71.39	67.27	78.03	72.62	64.73	79.96
ETP+VA	72.60	67.05	78.64	76.27	68.69	84.62	71.37	67.23	77.97	71.65	63.69	79.09
FEP	46.93	37.14	55.68	39.74	28.45	50.56	45.53	35.70	54.61	36.47	26.32	46.26
FEP+AN	62.35	56.33	68.10	65.12	56.49	73.40	62.74	57.43	67.58	60.87	52.30	69.11
FEP+LNZ	43.92	33.83	53.19	38.92	27.59	49.72	41.87	31.86	51.53	36.68	26.73	46.35
FEP+VA	43.52	33.29	52.83	38.89	27.74	49.73	41.83	31.88	51.48	35.96	25.84	45.65
FOS	72.59	66.04	79.79	75.29	66.08	84.89	71.03	66.41	78.22	69.99	59.89	79.36
MEM	72.61	67.13	78.68	80.45	72.60	89.10	73.13	68.10	81.24	72.59	64.80	79.98
MEM+AN	75.11	69.48	81.75	80.62	72.86	89.39	73.16	68.15	81.19	75.79	68.33	82.93
MEM+LNZ	74.24	68.69	80.72	80.60	72.81	89.31	73.14	68.16	81.31	74.73	67.13	81.98
MEM+VA	73.37	67.89	79.60	80.68	72.78	89.37	73.17	68.16	81.30	73.62	65.96	80.98
NIT	60.18	52.86	66.85	63.13	52.88	72.87	60.04	53.00	66.09	59.52	48.72	69.57
SXT	44.70	33.75	54.61	42.82	30.74	54.22	45.86	35.37	55.53	33.48	22.12	44.72
TZP	69.93	63.67	76.50	66.59	55.38	77.01	69.09	64.21	75.34	64.66	54.99	73.73
TZP+AN	61.99	55.84	67.71	63.74	54.96	72.09	61.42	55.64	66.58	61.59	53.02	69.75
TZP+LNZ	43.90	33.77	53.16	35.87	24.47	46.95	36.28	25.76	46.57	42.27	32.59	51.60
TZP+VA	43.48	33.33	52.83	35.86	24.47	46.93	36.29	25.77	46.60	41.61	31.82	50.80

	Previous hospitalization absent			Previous hospitalization present			Hipotension absent			Hipotension present		
**regimen**	**median_pct_reg**	**hdi_lower_pct_reg**	**hdi_upper_pct_reg**	**median_pct_reg**	**hdi_lower_pct_reg**	**hdi_upper_pct_reg**	**median_pct_reg**	**hdi_lower_pct_reg**	**hdi_upper_pct_reg**	**median_pct_reg**	**hdi_lower_pct_reg**	**hdi_upper_pct_reg**
AN	63.61	57.44	68.90	61.80	53.51	70.42	67.59	62.26	72.46	58.98	49.23	68.77
CAZ	43.90	32.92	53.85	35.24	25.90	44.17	47.58	37.44	56.44	29.83	19.22	40.36
CAZ+AN	61.62	55.09	67.20	59.16	51.15	67.48	66.56	61.17	71.49	52.27	42.70	61.74
CAZ+LNZ	45.01	34.25	54.57	36.15	26.85	45.03	47.89	37.89	56.68	33.46	22.83	43.61
CAZ+VA	45.00	34.39	54.63	35.61	26.20	44.41	47.90	37.85	56.67	32.42	21.79	42.54
CIP	26.07	14.70	38.58	30.44	20.90	39.78	34.81	23.21	46.14	23.92	13.33	35.07
CRO	45.37	34.77	54.90	32.64	23.12	41.72	47.30	37.26	56.33	29.01	18.43	39.56
CRO+AN	64.52	58.90	69.44	59.16	51.12	67.38	67.94	62.83	72.69	55.76	46.33	65.31
CRO+LNZ	45.46	34.96	55.07	33.54	24.18	42.58	47.56	37.54	56.43	30.14	19.59	40.74
CRO+VA	45.48	34.78	55.02	32.97	23.63	42.11	47.55	37.45	56.47	29.02	18.32	39.42
ETP	72.13	68.24	77.31	65.20	57.26	73.84	74.74	70.70	79.80	63.67	54.39	73.23
ETP+AN	74.10	69.82	80.73	70.04	61.88	79.21	77.13	72.76	83.01	68.56	59.22	78.09
ETP+LNZ	73.35	69.31	79.36	67.00	59.13	75.84	76.09	71.89	81.58	65.17	55.82	74.61
ETP+VA	73.34	69.28	79.30	66.09	58.17	74.83	76.09	71.91	81.56	63.64	54.21	72.86
FEP	45.33	34.48	55.14	36.32	26.97	45.19	48.62	38.56	57.39	32.66	21.71	43.27
FEP+AN	61.98	55.53	67.45	59.85	51.81	68.06	66.27	60.77	71.24	55.77	46.40	65.20
FEP+LNZ	41.45	30.22	51.89	36.14	26.81	45.01	46.20	35.86	55.39	31.30	20.60	41.70
FEP+VA	41.47	30.35	51.90	35.63	26.26	44.49	46.18	35.86	55.34	30.14	19.34	40.45
FOS	72.52	67.71	78.87	65.52	55.87	75.89	74.48	69.50	80.28	66.92	55.20	78.48
MEM	74.08	69.77	80.62	68.67	60.57	77.79	77.65	73.16	83.80	65.17	55.89	74.68
MEM+AN	74.99	70.30	82.31	71.19	62.98	80.71	78.43	73.66	84.95	68.53	59.25	78.23
MEM+LNZ	74.96	70.33	82.20	70.01	62.01	79.28	78.44	73.69	84.93	66.79	57.50	76.33
MEM+VA	75.01	70.34	82.35	68.96	60.94	78.01	78.42	73.62	84.97	65.13	55.84	74.59
NIT	60.14	52.02	66.85	56.27	46.83	65.52	64.37	57.59	70.37	53.34	42.04	64.31

SXT	48.44	37.21	58.26	32.56	22.36	42.41	46.95	35.86	56.76	36.11	23.85	47.84
TZP	67.35	61.60	72.65	64.93	55.08	75.23	72.16	66.78	77.64	57.91	46.12	69.19
TZP+AN	61.64	55.15	67.22	58.47	50.46	66.70	65.71	60.09	70.80	54.57	44.99	63.98
TZP+LNZ	44.02	33.16	53.88	31.35	21.87	40.44	44.06	33.48	53.68	33.44	22.86	43.69
TZP+VA	44.03	33.08	53.86	30.79	21.42	40.11	44.04	33.33	53.60	32.39	21.74	42.66

	Group 1			Group 2		
**regimen**	**median_pct_reg**	**hdi_lower_pct_reg**	**hdi_upper_pct_reg**	**median_pct_reg**	**hdi_lower_pct_reg**	**hdi_upper_pct_reg**
AN	76.02	56.13	81.18	75.72	56.28	80.31
CAZ	56.34	32.90	63.95	57.04	33.90	64.36
CAZ+AN	74.05	54.01	79.07	73.86	54.30	78.42
CAZ+LNZ	57.33	34.07	64.74	57.99	35.00	65.04
CAZ+VA	57.10	33.96	64.63	57.77	34.70	64.77
CIP	45.93	21.35	55.40	46.94	22.30	56.21
CRO	55.97	32.58	63.65	56.64	33.38	63.90
CRO+AN	75.71	55.92	80.71	75.44	56.07	79.88
CRO+LNZ	56.41	33.05	64.01	57.08	33.94	64.27
CRO+VA	56.14	32.76	63.75	56.87	33.71	64.10
ETP	82.78	63.00	88.51	81.91	62.99	86.80
ETP+AN	86.23	66.01	93.02	84.86	66.04	90.62
ETP+LNZ	84.33	64.45	90.50	83.24	64.40	88.47
ETP+VA	83.90	64.07	89.95	82.90	64.04	87.96
FEP	57.61	34.33	65.10	58.23	35.18	65.29
FEP+AN	74.45	54.49	79.52	74.26	54.75	78.79
FEP+LNZ	55.68	32.26	63.41	56.40	33.21	63.80
FEP+VA	55.43	31.89	63.15	56.16	32.85	63.57
FOS	83.91	63.24	90.78	83.24	63.50	89.19
MEM	85.62	65.53	92.25	84.35	65.47	89.88
MEM+AN	87.35	66.93	94.52	85.84	67.04	92.16
MEM+LNZ	86.78	66.39	93.72	85.34	66.51	91.31
MEM+VA	86.21	66.03	93.02	84.87	66.01	90.59
NIT	72.65	51.75	78.32	72.55	52.13	77.71
SXT	57.14	33.24	65.17	57.72	33.99	65.34
TZP	79.71	59.27	85.72	79.24	59.50	84.57
TZP+AN	73.85	53.79	78.92	73.69	54.06	78.23
TZP+LNZ	54.69	31.08	62.62	55.45	31.93	62.96
TZP+VA	54.45	30.81	62.45	55.20	31.67	62.79

Regimens ordered alphabetically

Table S11. $\hat{R}$ Index Values of WISCA model parameters in patients with community-onset urinary tract infections across sub-groups.

15-30 years		30-65 years		>65 years	
param	rhat	param	rhat	param	rhat
b_Intercept	1.0008	b_Intercept	1.0015	b_Intercept	1.0007
sd_pathogen__Intercept	1.0000	sd_pathogen__Intercept	1.0000	sd_pathogen__Intercept	1.0002
sd_regimen__Intercept	1.0000	sd_regimen__Intercept	1.0020	sd_regimen__Intercept	1.0002
Intercept	1.0008	Intercept	1.0015	Intercept	1.0007
r_pathogen[Escherichia.coli,Intercept]	1.0003	r_pathogen[Acinetobacter.baumannii,Intercept]	1.0003	r_pathogen[Candida.glabrata,Intercept]	1.0001
r_pathogen[Klebsiella.pneumoniae,Intercept]	1.0001	r_pathogen[Candida.glabrata,Intercept]	1.0002	r_pathogen[Candida.tropicalis,Intercept]	1.0001
r_pathogen[Proteus.mirabilis,Intercept]	1.0000	r_pathogen[Candida.parapsilosis,Intercept]	1.0004	r_pathogen[Entero.faecalis,Intercept]	1.0001
r_regimen[AN,Intercept]	1.0001	r_pathogen[Citrobacter.freundii,Intercept]	1.0007	r_pathogen[Entero.faecium,Intercept]	1.0002
r_regimen[CAZ,Intercept]	1.0002	r_pathogen[Entero.faecalis,Intercept]	1.0007	r_pathogen[Escherichia.coli,Intercept]	1.0002
r_regimen[CAZ+AN,Intercept]	1.0000	r_pathogen[Enterobacter.cloacae,Intercept]	1.0004	r_pathogen[Klebsiella.pneumoniae,Intercept]	1.0003
r_regimen[CAZ+LNZ,Intercept]	1.0003	r_pathogen[Escherichia.coli,Intercept]	1.0011	r_pathogen[Proteus.mirabilis,Intercept]	1.0000
r_regimen[CAZ+VA,Intercept]	1.0001	r_pathogen[Klebsiella.pneumoniae,Intercept]	1.0009	r_pathogen[Pseudomonas.aeruginosa,Intercept]	1.0002
r_regimen[CIP,Intercept]	1.0003	r_pathogen[Morganella.morganii,Intercept]	1.0002	r_regimen[AN,Intercept]	1.0004
r_regimen[CRO,Intercept]	1.0005	r_pathogen[Proteus.mirabilis,Intercept]	1.0010	r_regimen[CAZ,Intercept]	1.0004
r_regimen[CRO+AN,Intercept]	1.0002	r_pathogen[Pseudomonas.aeruginosa,Intercept]	1.0010	r_regimen[CAZ+AN,Intercept]	1.0006
r_regimen[CRO+LNZ,Intercept]	1.0002	r_pathogen[Staphylococcus.aureus,Intercept]	1.0008	r_regimen[CAZ+LNZ,Intercept]	1.0002
r_regimen[CRO+VA,Intercept]	1.0002	r_pathogen[Staphylococcus.saprophyticus,Intercept]	1.0003	r_regimen[CAZ+VA,Intercept]	1.0006
r_regimen[ETP,Intercept]	1.0001	r_regimen[AN,Intercept]	1.0006	r_regimen[CIP,Intercept]	1.0005
r_regimen[ETP+AN,Intercept]	1.0000	r_regimen[CAZ,Intercept]	1.0007	r_regimen[CRO,Intercept]	1.0001
r_regimen[ETP+LNZ,Intercept]	1.0001	r_regimen[CAZ+AN,Intercept]	1.0008	r_regimen[CRO+AN,Intercept]	1.0007
r_regimen[ETP+VA,Intercept]	1.0003	r_regimen[CAZ+LNZ,Intercept]	1.0008	r_regimen[CRO+LNZ,Intercept]	1.0005

r_regimen[FEP,Intercept]	1.0002	r_regimen[CAZ+VA,Intercept]	1.0010	r_regimen[CRO+VA,Intercept]	1.0006
r_regimen[FEP+AN,Intercept]	1.0001	r_regimen[CIP,Intercept]	1.0007	r_regimen[ETP,Intercept]	1.0003
r_regimen[FEP+LNZ,Intercept]	1.0003	r_regimen[CRO,Intercept]	1.0007	r_regimen[ETP+AN,Intercept]	1.0003
r_regimen[FEP+VA,Intercept]	1.0004	r_regimen[CRO+AN,Intercept]	1.0007	r_regimen[ETP+LNZ,Intercept]	1.0001
r_regimen[FOS,Intercept]	1.0003	r_regimen[CRO+LNZ,Intercept]	1.0008	r_regimen[ETP+VA,Intercept]	1.0002
r_regimen[MEM,Intercept]	1.0002	r_regimen[CRO+VA,Intercept]	1.0010	r_regimen[FEP,Intercept]	1.0006
r_regimen[MEM+AN,Intercept]	1.0005	r_regimen[ETP,Intercept]	1.0002	r_regimen[FEP+AN,Intercept]	1.0004
r_regimen[MEM+LNZ,Intercept]	1.0001	r_regimen[ETP+AN,Intercept]	1.0004	r_regimen[FEP+LNZ,Intercept]	1.0004
r_regimen[MEM+VA,Intercept]	1.0001	r_regimen[ETP+LNZ,Intercept]	1.0005	r_regimen[FEP+VA,Intercept]	1.0005
r_regimen[NIT,Intercept]	1.0002	r_regimen[ETP+VA,Intercept]	1.0004	r_regimen[FOS,Intercept]	1.0001
r_regimen[SXT,Intercept]	1.0001	r_regimen[FEP,Intercept]	1.0009	r_regimen[MEM,Intercept]	1.0003
r_regimen[TZP,Intercept]	1.0004	r_regimen[FEP+AN,Intercept]	1.0010	r_regimen[MEM+AN,Intercept]	1.0002
r_regimen[TZP+AN,Intercept]	1.0002	r_regimen[FEP+LNZ,Intercept]	1.0012	r_regimen[MEM+LNZ,Intercept]	1.0003
r_regimen[TZP+LNZ,Intercept]	1.0003	r_regimen[FEP+VA,Intercept]	1.0008	r_regimen[MEM+VA,Intercept]	1.0002
r_regimen[TZP+VA,Intercept]	1.0001	r_regimen[FOS,Intercept]	1.0003	r_regimen[NIT,Intercept]	1.0004
lprior	1.0001	r_regimen[MEM,Intercept]	1.0004	r_regimen[SXT,Intercept]	1.0002
lp__	1.0002	r_regimen[MEM+AN,Intercept]	1.0005	r_regimen[TZP,Intercept]	1.0002
		r_regimen[MEM+LNZ,Intercept]	1.0005	r_regimen[TZP+AN,Intercept]	1.0005
		r_regimen[MEM+VA,Intercept]	1.0003	r_regimen[TZP+LNZ,Intercept]	1.0006
		r_regimen[NIT,Intercept]	1.0005	r_regimen[TZP+VA,Intercept]	1.0007
		r_regimen[SXT,Intercept]	1.0007	lprior	1.0001
		r_regimen[TZP,Intercept]	1.0003	lp__	1.0001
		r_regimen[TZP+AN,Intercept]	1.0006		
		r_regimen[TZP+LNZ,Intercept]	1.0008		
		r_regimen[TZP+VA,Intercept]	1.0009		
		lprior	1.0005		
		lp__	1.0004		

Non-recurrent UTI		Recurrent UTI		Antibiotic treatment < 90 days absent		Antibiotic treatment < 90 days present	
param	rhat	param	rhat	param	rhat	param	rhat
b_Intercept	1.0007	b_Intercept	1.0013	b_Intercept	1.0007	b_Intercept	1.0010

sd_pathogen__Intercept	1.0001	sd_pathogen__Intercept	1.0001	sd_pathogen__Intercept	1.0002	sd_pathogen__Intercept	1.0004
sd_regimen__Intercept	1.0007	sd_regimen__Intercept	1.0004	sd_regimen__Intercept	1.0003	sd_regimen__Intercept	1.0005
Intercept	1.0007	Intercept	1.0013	Intercept	1.0007	Intercept	1.0010
r_pathogen[Candida.glabrata,Intercept]	1.0003	r_pathogen[Acinetobacter.baumannii,Intercept]	1.0001	r_pathogen[Acinetobacter.baumannii,Intercept]	1.0002	r_pathogen[Candida.glabrata,Intercept]	1.0001
r_pathogen[Candida.parapsilosis,Intercept]	1.0003	r_pathogen[Candida.glabrata,Intercept]	1.0001	r_pathogen[Candida.glabrata,Intercept]	1.0001	r_pathogen[Candida.tropicalis,Intercept]	1.0005
r_pathogen[Candida.tropicalis,Intercept]	1.0001	r_pathogen[Citrobacter.freundii,Intercept]	1.0004	r_pathogen[Candida.parapsilosis,Intercept]	1.0000	r_pathogen[Citrobacter.freundii,Intercept]	1.0009
r_pathogen[Citrobacter.freundii,Intercept]	1.0009	r_pathogen[Entero.faecalis,Intercept]	1.0005	r_pathogen[Entero.faecalis,Intercept]	1.0002	r_pathogen[Entero.faecium,Intercept]	1.0006
r_pathogen[Entero.faecalis,Intercept]	1.0011	r_pathogen[Escherichia.coli,Intercept]	1.0006	r_pathogen[Enterobacter.cloacae,Intercept]	1.0001	r_pathogen[Escherichia.coli,Intercept]	1.0010
r_pathogen[Entero.faecium,Intercept]	1.0006	r_pathogen[Klebsiella.pneumoniae,Intercept]	1.0004	r_pathogen[Escherichia.coli,Intercept]	1.0005	r_pathogen[Klebsiella.pneumoniae,Intercept]	1.0010
r_pathogen[Enterobacter.cloacae,Intercept]	1.0006	r_pathogen[Proteus.mirabilis,Intercept]	1.0004	r_pathogen[Klebsiella.pneumoniae,Intercept]	1.0004	r_pathogen[Morganella.morganii,Intercept]	1.0004
r_pathogen[Escherichia.coli,Intercept]	1.0015	r_pathogen[Pseudomonas.aeruginosa,Intercept]	1.0007	r_pathogen[Proteus.mirabilis,Intercept]	1.0004	r_pathogen[Proteus.mirabilis,Intercept]	1.0006
r_pathogen[Klebsiella.pneumoniae,Intercept]	1.0014	r_pathogen[Staphylococcus.aureus,Intercept]	1.0004	r_pathogen[Pseudomonas.aeruginosa,Intercept]	1.0003	r_pathogen[Pseudomonas.aeruginosa, Intercept]	1.0009
r_pathogen[Morganella.morganii,Intercept]	1.0006	r_pathogen[Staphylococcus.saprophyticus,Intercept]	1.0002	r_pathogen[Staphylococcus.aureus,Intercept]	1.0002	r_pathogen[Staphylococcus.aureus,Intercept]	1.0006
r_pathogen[Proteus.mirabilis,Intercept]	1.0012	r_regimen[AN,Intercept]	1.0004	r_regimen[AN,Intercept]	1.0006	r_pathogen[Staphylococcus.saprophyticus,Intercept]	1.0004
r_pathogen[Pseudomonas.aeruginosa,Intercept]	1.0014	r_regimen[CAZ,Intercept]	1.0003	r_regimen[CAZ,Intercept]	1.0010	r_regimen[AN,Intercept]	1.0001
r_pathogen[Staphylococcus.aureus,Intercept]	1.0006	r_regimen[CAZ+AN,Intercept]	1.0004	r_regimen[CAZ+AN,Intercept]	1.0007	r_regimen[CAZ,Intercept]	1.0001
r_regimen[AN,Intercept]	1.0010	r_regimen[CAZ+LNZ,Intercept]	1.0004	r_regimen[CAZ+LNZ,Intercept]	1.0010	r_regimen[CAZ+AN,Intercept]	1.0002
r_regimen[CAZ,Intercept]	1.0016	r_regimen[CAZ+VA,Intercept]	1.0005	r_regimen[CAZ+VA,Intercept]	1.0008	r_regimen[CAZ+LNZ,Intercept]	1.0002
r_regimen[CAZ+AN,Intercept]	1.0011	r_regimen[CIP,Intercept]	1.0004	r_regimen[CIP,Intercept]	1.0009	r_regimen[CAZ+VA,Intercept]	1.0003
r_regimen[CAZ+LNZ,Intercept]	1.0015	r_regimen[CRO,Intercept]	1.0006	r_regimen[CRO,Intercept]	1.0008	r_regimen[CIP,Intercept]	1.0003
r_regimen[CAZ+VA,Intercept]	1.0013	r_regimen[CRO+AN,Intercept]	1.0004	r_regimen[CRO+AN,Intercept]	1.0006	r_regimen[CRO,Intercept]	1.0002
r_regimen[CIP,Intercept]	1.0011	r_regimen[CRO+LNZ,Intercept]	1.0006	r_regimen[CRO+LNZ,Intercept]	1.0009	r_regimen[CRO+AN,Intercept]	1.0002
r_regimen[CRO,Intercept]	1.0017	r_regimen[CRO+VA,Intercept]	1.0007	r_regimen[CRO+VA,Intercept]	1.0008	r_regimen[CRO+LNZ,Intercept]	1.0002

r_regimen[CRO+AN,Intercept]	1.0010	r_regimen[ETP,Intercept]	1.0002	r_regimen[ETP,Intercept]	1.0004	r_regimen[CRO+VA,Intercept]	1.0001
r_regimen[CRO+LNZ,Intercept]	1.0014	r_regimen[ETP+AN,Intercept]	1.0004	r_regimen[ETP+AN,Intercept]	1.0002	r_regimen[ETP,Intercept]	1.0002
r_regimen[CRO+VA,Intercept]	1.0014	r_regimen[ETP+LNZ,Intercept]	1.0002	r_regimen[ETP+LNZ,Intercept]	1.0004	r_regimen[ETP+AN,Intercept]	1.0001
r_regimen[ETP,Intercept]	1.0006	r_regimen[ETP+VA,Intercept]	1.0003	r_regimen[ETP+VA,Intercept]	1.0007	r_regimen[ETP+LNZ,Intercept]	1.0001
r_regimen[ETP+AN,Intercept]	1.0006	r_regimen[FEP,Intercept]	1.0005	r_regimen[FEP,Intercept]	1.0007	r_regimen[ETP+VA,Intercept]	1.0002
r_regimen[ETP+LNZ,Intercept]	1.0005	r_regimen[FEP+AN,Intercept]	1.0005	r_regimen[FEP+AN,Intercept]	1.0008	r_regimen[FEP,Intercept]	1.0002
r_regimen[ETP+VA,Intercept]	1.0005	r_regimen[FEP+LNZ,Intercept]	1.0004	r_regimen[FEP+LNZ,Intercept]	1.0007	r_regimen[FEP+AN,Intercept]	1.0001
r_regimen[FEP,Intercept]	1.0013	r_regimen[FEP+VA,Intercept]	1.0006	r_regimen[FEP+VA,Intercept]	1.0006	r_regimen[FEP+LNZ,Intercept]	1.0002
r_regimen[FEP+AN,Intercept]	1.0011	r_regimen[FOS,Intercept]	1.0003	r_regimen[FOS,Intercept]	1.0001	r_regimen[FEP+VA,Intercept]	1.0002
r_regimen[FEP+LNZ,Intercept]	1.0016	r_regimen[MEM,Intercept]	1.0001	r_regimen[MEM,Intercept]	1.0003	r_regimen[FOS,Intercept]	1.0001
r_regimen[FEP+VA,Intercept]	1.0012	r_regimen[MEM+AN,Intercept]	1.0001	r_regimen[MEM+AN,Intercept]	1.0001	r_regimen[MEM,Intercept]	1.0001
r_regimen[FOS,Intercept]	1.0004	r_regimen[MEM+LNZ,Intercept]	1.0001	r_regimen[MEM+LNZ,Intercept]	1.0003	r_regimen[MEM+AN,Intercept]	1.0003
r_regimen[MEM,Intercept]	1.0006	r_regimen[MEM+VA,Intercept]	1.0001	r_regimen[MEM+VA,Intercept]	1.0004	r_regimen[MEM+LNZ,Intercept]	1.0000
r_regimen[MEM+AN,Intercept]	1.0008	r_regimen[NIT,Intercept]	1.0001	r_regimen[NIT,Intercept]	1.0006	r_regimen[MEM+VA,Intercept]	1.0001
r_regimen[MEM+LNZ,Intercept]	1.0007	r_regimen[SXT,Intercept]	1.0003	r_regimen[SXT,Intercept]	1.0008	r_regimen[NIT,Intercept]	1.0004
r_regimen[MEM+VA,Intercept]	1.0005	r_regimen[TZP,Intercept]	1.0003	r_regimen[TZP,Intercept]	1.0004	r_regimen[SXT,Intercept]	1.0001
r_regimen[NIT,Intercept]	1.0007	r_regimen[TZP+AN,Intercept]	1.0005	r_regimen[TZP+AN,Intercept]	1.0008	r_regimen[TZP,Intercept]	1.0001
r_regimen[SXT,Intercept]	1.0011	r_regimen[TZP+LNZ,Intercept]	1.0007	r_regimen[TZP+LNZ,Intercept]	1.0010	r_regimen[TZP+AN,Intercept]	1.0002
r_regimen[TZP,Intercept]	1.0007	r_regimen[TZP+VA,Intercept]	1.0004	r_regimen[TZP+VA,Intercept]	1.0011	r_regimen[TZP+LNZ,Intercept]	1.0000
r_regimen[TZP+AN,Intercept]	1.0010	lprior	1.0000	lprior	1.0004	r_regimen[TZP+VA,Intercept]	1.0000
r_regimen[TZP+LNZ,Intercept]	1.0013	lp__	1.0003	lp__	1.0005	lprior	1.0004
r_regimen[TZP+VA,Intercept]	1.0013					lp__	1.0001
lprior	1.0001						
lp__	1.0004						

Previous hospitalization absent		Previous hospitalization present		Hipotension absent		Hipotension present	
param	rhat	param	rhat	param	rhat	param	rhat
b_Intercept	1.0011	b_Intercept	1.0008	b_Intercept	1.0013	b_Intercept	1.0004
sd_pathogen__Intercept	1.0001	sd_pathogen__Intercept	1.0000	sd_pathogen__Intercept	1.0002	sd_pathogen__Intercept	1.0001
sd_regimen__Intercept	1.0009	sd_regimen__Intercept	1.0010	sd_regimen__Intercept	1.0007	sd_regimen__Intercept	1.0004
Intercept	1.0011	Intercept	1.0008	Intercept	1.0013	Intercept	1.0004
r_pathogen[Candida.glabrata,Intercept]	1.0002	r_pathogen[Acinetobacter.baumannii,Intercept]	1.0000	r_pathogen[Acinetobacter.baumannii,Intercept]	1.0004	r_pathogen[Candida.glabrata,Intercept]	1.0001
r_pathogen[Candida.parapsilosis,Intercept]	1.0004	r_pathogen[Candida.glabrata,Intercept]	1.0000	r_pathogen[Candida.glabrata,Intercept]	1.0005	r_pathogen[Candida.tropicalis,Intercept]	1.0003
r_pathogen[Candida.tropicalis,Intercept]	1.0002	r_pathogen[Candida.tropicalis,Intercept]	1.0003	r_pathogen[Candida.parapsilosis,Intercept]	1.0006	r_pathogen[Entero.faecalis,Intercept]	1.0005
r_pathogen[Entero.faecalis,Intercept]	1.0005	r_pathogen[Citrobacter.freundii,Intercept]	1.0005	r_pathogen[Citrobacter.freundii,Intercept]	1.0008	r_pathogen[Entero.faecium,Intercept]	1.0005
r_pathogen[Enterobacter.cloacae,Intercept]	1.0003	r_pathogen[Entero.faecalis,Intercept]	1.0001	r_pathogen[Entero.faecalis,Intercept]	1.0006	r_pathogen[Escherichia.coli,Intercept]	1.0009
r_pathogen[Escherichia.coli,Intercept]	1.0007	r_pathogen[Entero.faecium,Intercept]	1.0005	r_pathogen[Enterobacter.cloacae,Intercept]	1.0007	r_pathogen[Klebsiella.pneumoniae,Intercept]	1.0009
r_pathogen[Klebsiella.pneumoniae,Intercept]	1.0006	r_pathogen[Escherichia.coli,Intercept]	1.0009	r_pathogen[Escherichia.coli,Intercept]	1.0014	r_pathogen[Pseudomonas.aeruginosa,Intercept]	1.0008
r_pathogen[Proteus.mirabilis,Intercept]	1.0006	r_pathogen[Klebsiella.pneumoniae,Intercept]	1.0007	r_pathogen[Klebsiella.pneumoniae,Intercept]	1.0014	r_pathogen[Staphylococcus.aureus,Intercept]	1.0003
r_pathogen[Pseudomonas.aeruginosa,Intercept]	1.0005	r_pathogen[Morganella.morganii,Intercept]	1.0003	r_pathogen[Morganella.morganii,Intercept]	1.0004	r_regimen[AN,Intercept]	1.0004
r_pathogen[Staphylococcus.aureus,Intercept]	1.0004	r_pathogen[Proteus.mirabilis,Intercept]	1.0005	r_pathogen[Proteus.mirabilis,Intercept]	1.0013	r_regimen[CAZ,Intercept]	1.0003
r_pathogen[Staphylococcus.saprophyticus,Intercept]	1.0003	r_pathogen[Pseudomonas.aeruginosa,Intercept]	1.0006	r_pathogen[Pseudomonas.aeruginosa,Intercept]	1.0015	r_regimen[CAZ+AN,Intercept]	1.0005
r_regimen[AN,Intercept]	1.0016	r_pathogen[Staphylococcus.aureus,Intercept]	1.0005	r_pathogen[Staphylococcus.aureus,Intercept]	1.0010	r_regimen[CAZ+LNZ,Intercept]	1.0004
r_regimen[CAZ,Intercept]	1.0021	r_regimen[AN,Intercept]	1.0002	r_pathogen[Staphylococcus.saprophyticus,Intercept]	1.0003	r_regimen[CAZ+VA,Intercept]	1.0006
r_regimen[CAZ+AN,Intercept]	1.0014	r_regimen[CAZ,Intercept]	1.0008	r_regimen[AN,Intercept]	1.0008	r_regimen[CIP,Intercept]	1.0003
r_regimen[CAZ+LNZ,Intercept]	1.0020	r_regimen[CAZ+AN,Intercept]	1.0004	r_regimen[CAZ,Intercept]	1.0009	r_regimen[CRO,Intercept]	1.0005
r_regimen[CAZ+VA,Intercept]	1.0019	r_regimen[CAZ+LNZ,Intercept]	1.0005	r_regimen[CAZ+AN,Intercept]	1.0008	r_regimen[CRO+AN,Intercept]	1.0006
r_regimen[CIP,Intercept]	1.0017	r_regimen[CAZ+VA,Intercept]	1.0007	r_regimen[CAZ+LNZ,Intercept]	1.0010	r_regimen[CRO+LNZ,Intercept]	1.0005
r_regimen[CRO,Intercept]	1.0022	r_regimen[CIP,Intercept]	1.0009	r_regimen[CAZ+VA,Intercept]	1.0010	r_regimen[CRO+VA,Intercept]	1.0005

r_regimen[CRO+AN,Intercept]	1.0018	r_regimen[CRO,Intercept]	1.0006	r_regimen[CIP,Intercept]	1.0008	r_regimen[ETP,Intercept]	1.0004
r_regimen[CRO+LNZ,Intercept]	1.0017	r_regimen[CRO+AN,Intercept]	1.0002	r_regimen[CRO,Intercept]	1.0008	r_regimen[ETP+AN,Intercept]	1.0000
r_regimen[CRO+VA,Intercept]	1.0018	r_regimen[CRO+LNZ,Intercept]	1.0006	r_regimen[CRO+AN,Intercept]	1.0009	r_regimen[ETP+LNZ,Intercept]	1.0004
r_regimen[ETP,Intercept]	1.0011	r_regimen[CRO+VA,Intercept]	1.0006	r_regimen[CRO+LNZ,Intercept]	1.0007	r_regimen[ETP+VA,Intercept]	1.0003
r_regimen[ETP+AN,Intercept]	1.0006	r_regimen[ETP,Intercept]	1.0005	r_regimen[CRO+VA,Intercept]	1.0007	r_regimen[FEP,Intercept]	1.0006
r_regimen[ETP+LNZ,Intercept]	1.0005	r_regimen[ETP+AN,Intercept]	1.0002	r_regimen[ETP,Intercept]	1.0003	r_regimen[FEP+AN,Intercept]	1.0003
r_regimen[ETP+VA,Intercept]	1.0011	r_regimen[ETP+LNZ,Intercept]	1.0002	r_regimen[ETP+AN,Intercept]	1.0006	r_regimen[FEP+LNZ,Intercept]	1.0003
r_regimen[FEP,Intercept]	1.0018	r_regimen[ETP+VA,Intercept]	1.0006	r_regimen[ETP+LNZ,Intercept]	1.0004	r_regimen[FEP+VA,Intercept]	1.0006
r_regimen[FEP+AN,Intercept]	1.0017	r_regimen[FEP,Intercept]	1.0006	r_regimen[ETP+VA,Intercept]	1.0003	r_regimen[FOS,Intercept]	1.0003
r_regimen[FEP+LNZ,Intercept]	1.0017	r_regimen[FEP+AN,Intercept]	1.0003	r_regimen[FEP,Intercept]	1.0007	r_regimen[MEM,Intercept]	1.0002
r_regimen[FEP+VA,Intercept]	1.0018	r_regimen[FEP+LNZ,Intercept]	1.0007	r_regimen[FEP+AN,Intercept]	1.0008	r_regimen[MEM+AN,Intercept]	1.0003
r_regimen[FOS,Intercept]	1.0001	r_regimen[FEP+VA,Intercept]	1.0005	r_regimen[FEP+LNZ,Intercept]	1.0009	r_regimen[MEM+LNZ,Intercept]	1.0002
r_regimen[MEM,Intercept]	1.0004	r_regimen[FOS,Intercept]	1.0002	r_regimen[FEP+VA,Intercept]	1.0008	r_regimen[MEM+VA,Intercept]	1.0003
r_regimen[MEM+AN,Intercept]	1.0006	r_regimen[MEM,Intercept]	1.0003	r_regimen[FOS,Intercept]	1.0004	r_regimen[NIT,Intercept]	1.0002
r_regimen[MEM+LNZ,Intercept]	1.0008	r_regimen[MEM+AN,Intercept]	1.0001	r_regimen[MEM,Intercept]	1.0004	r_regimen[SXT,Intercept]	1.0005
r_regimen[MEM+VA,Intercept]	1.0007	r_regimen[MEM+LNZ,Intercept]	1.0005	r_regimen[MEM+AN,Intercept]	1.0004	r_regimen[TZP,Intercept]	1.0002
r_regimen[NIT,Intercept]	1.0015	r_regimen[MEM+VA,Intercept]	1.0006	r_regimen[MEM+LNZ,Intercept]	1.0003	r_regimen[TZP+AN,Intercept]	1.0004
r_regimen[SXT,Intercept]	1.0015	r_regimen[NIT,Intercept]	1.0005	r_regimen[MEM+VA,Intercept]	1.0004	r_regimen[TZP+LNZ,Intercept]	1.0005
r_regimen[TZP,Intercept]	1.0007	r_regimen[SXT,Intercept]	1.0003	r_regimen[NIT,Intercept]	1.0004	r_regimen[TZP+VA,Intercept]	1.0003
r_regimen[TZP+AN,Intercept]	1.0017	r_regimen[TZP,Intercept]	1.0005	r_regimen[SXT,Intercept]	1.0007	lprior	1.0002
r_regimen[TZP+LNZ,Intercept]	1.0018	r_regimen[TZP+AN,Intercept]	1.0005	r_regimen[TZP,Intercept]	1.0006	lp__	1.0011
r_regimen[TZP+VA,Intercept]	1.0019	r_regimen[TZP+LNZ,Intercept]	1.0006	r_regimen[TZP+AN,Intercept]	1.0009		
lprior	1.0003	r_regimen[TZP+VA,Intercept]	1.0003	r_regimen[TZP+LNZ,Intercept]	1.0009		
lp__	1.0008	lprior	1.0004	r_regimen[TZP+VA,Intercept]	1.0009		

		lp__	1.0008	lprior	1.0002		
				lp__	1.0004		

Gruoup 1		Gruoup 2	
param	rhat	param	rhat
b_Intercept	1.0010	b_Intercept	1.0010
sd_pathogen__Intercept	1.0000	sd_pathogen__Intercept	1.0000
sd_regimen__Intercept	1.0005	sd_regimen__Intercept	1.0005
Intercept	1.0010	Intercept	1.0010
r_pathogen[Acinetobacter.baumannii,Intercept]	1.0006	r_pathogen[Acinetobacter.baumannii,Intercept]	1.0006
r_pathogen[Candida.glabrata,Intercept]	1.0001	r_pathogen[Candida.glabrata,Intercept]	1.0001
r_pathogen[Candida.parapsilosis,Intercept]	1.0004	r_pathogen[Candida.parapsilosis,Intercept]	1.0004
r_pathogen[Candida.tropicalis,Intercept]	1.0001	r_pathogen[Candida.tropicalis,Intercept]	1.0001
r_pathogen[Citrobacter.freundii,Intercept]	1.0009	r_pathogen[Citrobacter.freundii,Intercept]	1.0009
r_pathogen[Entero.faecalis,Intercept]	1.0006	r_pathogen[Entero.faecalis,Intercept]	1.0006
r_pathogen[Entero.faecium,Intercept]	1.0007	r_pathogen[Entero.faecium,Intercept]	1.0007
r_pathogen[Enterobacter.cloacae,Intercept]	1.0006	r_pathogen[Enterobacter.cloacae,Intercept]	1.0006
r_pathogen[Escherichia.coli,Intercept]	1.0008	r_pathogen[Escherichia.coli,Intercept]	1.0008
r_pathogen[Klebsiella.pneumoniae,Intercept]	1.0008	r_pathogen[Klebsiella.pneumoniae,Intercept]	1.0008
r_pathogen[Morganella.morganii,Intercept]	1.0004	r_pathogen[Morganella.morganii,Intercept]	1.0004
r_pathogen[Proteus.mirabilis,Intercept]	1.0008	r_pathogen[Proteus.mirabilis,Intercept]	1.0008
r_pathogen[Pseudomonas.aeruginosa,Intercept]	1.0008	r_pathogen[Pseudomonas.aeruginosa,Intercept]	1.0008
r_pathogen[Staphylococcus.aureus,Intercept]	1.0007	r_pathogen[Staphylococcus.aureus,Intercept]	1.0007
r_pathogen[Staphylococcus.saprophyticus,Intercept]	1.0004	r_pathogen[Staphylococcus.saprophyticus,Intercept]	1.0004
r_regimen[AN,Intercept]	1.0004	r_regimen[AN,Intercept]	1.0004
r_regimen[CAZ,Intercept]	1.0004	r_regimen[CAZ,Intercept]	1.0004

r_regimen[CAZ+AN,Intercept]	1.0005	r_regimen[CAZ+AN,Intercept]	1.0005
r_regimen[CAZ+LNZ,Intercept]	1.0006	r_regimen[CAZ+LNZ,Intercept]	1.0006
r_regimen[CAZ+VA,Intercept]	1.0006	r_regimen[CAZ+VA,Intercept]	1.0006
r_regimen[CIP,Intercept]	1.0005	r_regimen[CIP,Intercept]	1.0005
r_regimen[CRO,Intercept]	1.0006	r_regimen[CRO,Intercept]	1.0006
r_regimen[CRO+AN,Intercept]	1.0006	r_regimen[CRO+AN,Intercept]	1.0006
r_regimen[CRO+LNZ,Intercept]	1.0006	r_regimen[CRO+LNZ,Intercept]	1.0006
r_regimen[CRO+VA,Intercept]	1.0005	r_regimen[CRO+VA,Intercept]	1.0005
r_regimen[ETP,Intercept]	1.0005	r_regimen[ETP,Intercept]	1.0005
r_regimen[ETP+AN,Intercept]	1.0002	r_regimen[ETP+AN,Intercept]	1.0002
r_regimen[ETP+LNZ,Intercept]	1.0005	r_regimen[ETP+LNZ,Intercept]	1.0005
r_regimen[ETP+VA,Intercept]	1.0004	r_regimen[ETP+VA,Intercept]	1.0004
r_regimen[FEP,Intercept]	1.0006	r_regimen[FEP,Intercept]	1.0006
r_regimen[FEP+AN,Intercept]	1.0004	r_regimen[FEP+AN,Intercept]	1.0004
r_regimen[FEP+LNZ,Intercept]	1.0005	r_regimen[FEP+LNZ,Intercept]	1.0005
r_regimen[FEP+VA,Intercept]	1.0005	r_regimen[FEP+VA,Intercept]	1.0005
r_regimen[FOS,Intercept]	1.0002	r_regimen[FOS,Intercept]	1.0002
r_regimen[MEM,Intercept]	1.0001	r_regimen[MEM,Intercept]	1.0001
r_regimen[MEM+AN,Intercept]	1.0002	r_regimen[MEM+AN,Intercept]	1.0002
r_regimen[MEM+LNZ,Intercept]	1.0004	r_regimen[MEM+LNZ,Intercept]	1.0004
r_regimen[MEM+VA,Intercept]	1.0002	r_regimen[MEM+VA,Intercept]	1.0002
r_regimen[NIT,Intercept]	1.0006	r_regimen[NIT,Intercept]	1.0006
r_regimen[SXT,Intercept]	1.0004	r_regimen[SXT,Intercept]	1.0004
r_regimen[TZP,Intercept]	1.0004	r_regimen[TZP,Intercept]	1.0004

r_regimen[TZP+AN,Intercept]	1.0005	r_regimen[TZP+AN,Intercept]	1.0005
r_regimen[TZP+LNZ,Intercept]	1.0005	r_regimen[TZP+LNZ,Intercept]	1.0005
r_regimen[TZP+VA,Intercept]	1.0008	r_regimen[TZP+VA,Intercept]	1.0008
lprior	1.0001	lprior	1.0001
lp__	1.0004	lp__	1.0004

Supplementary Figures

Figure S1. Monte Carlo Markov Chains (MCMC) traceplots of the CoUTI WISCA model parameters.

Figure S2. Density plots of the posterior distributions of the CoUTI WISCA model parameters.

Figure S3. Autocorrelation plots for distal lags ($h \geq 2$) in posterior samples of the CoUTI WISCA model parameters.

Figure S1. Monte Carlo Markov Chains (MCMC) traceplots of the CoUTI WISCA model parameters.

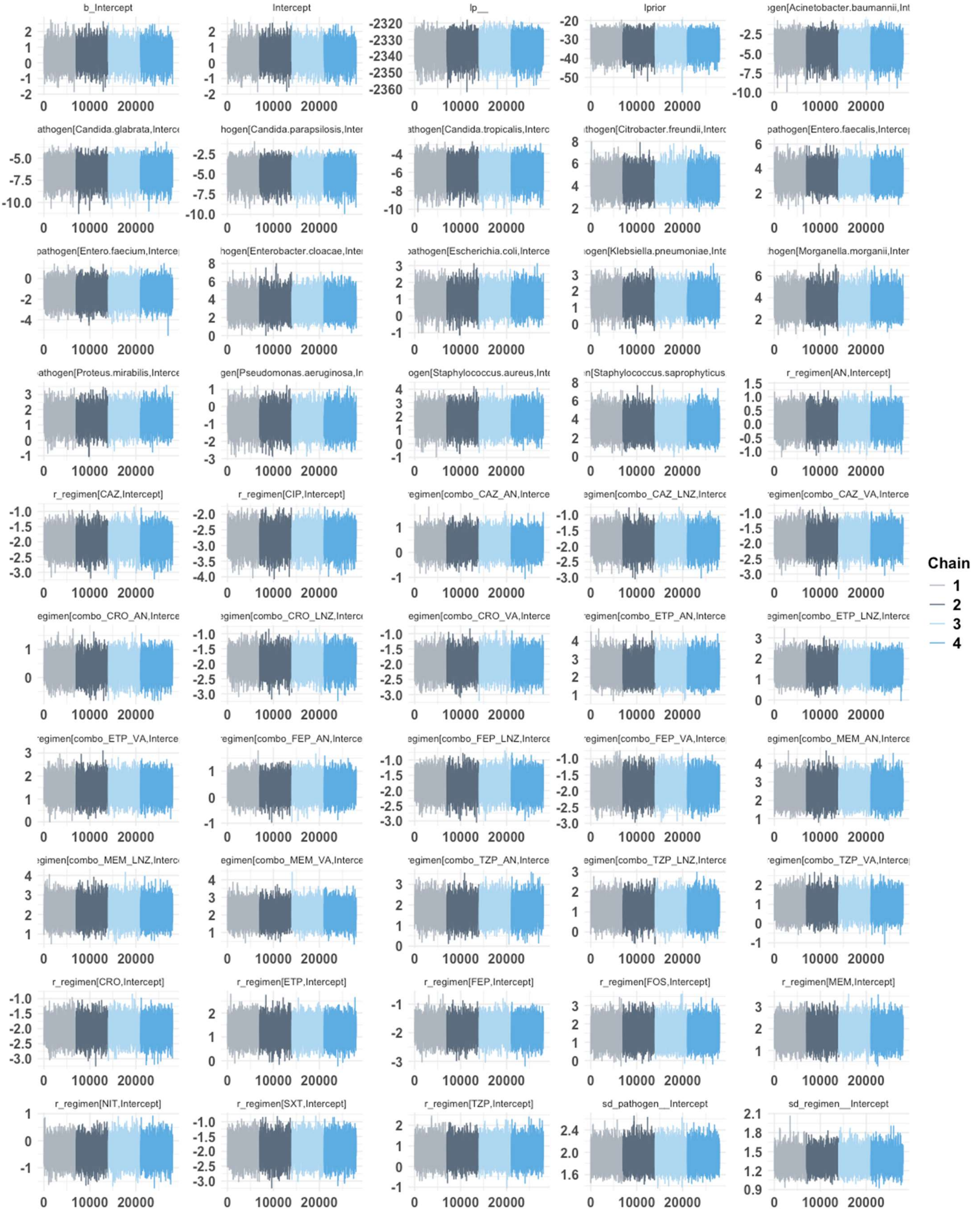

Figure S2. Density plots of the posterior distributions of the CoUTI WISCA model parameters.

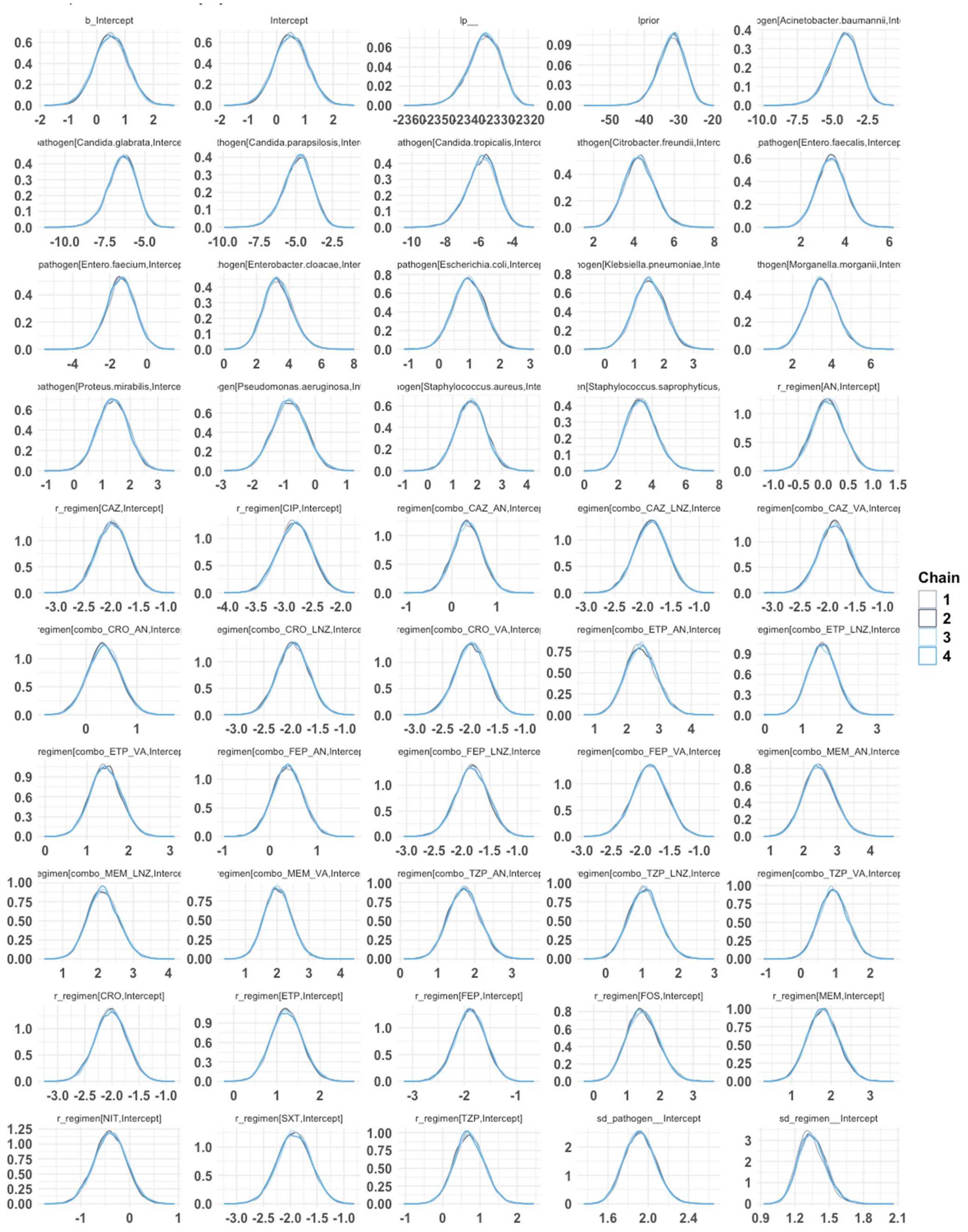

Figure S3. Autocorrelation plots for distal lags (h ≥ 2) in posterior samples of the CoUTI WISCA model parameters.

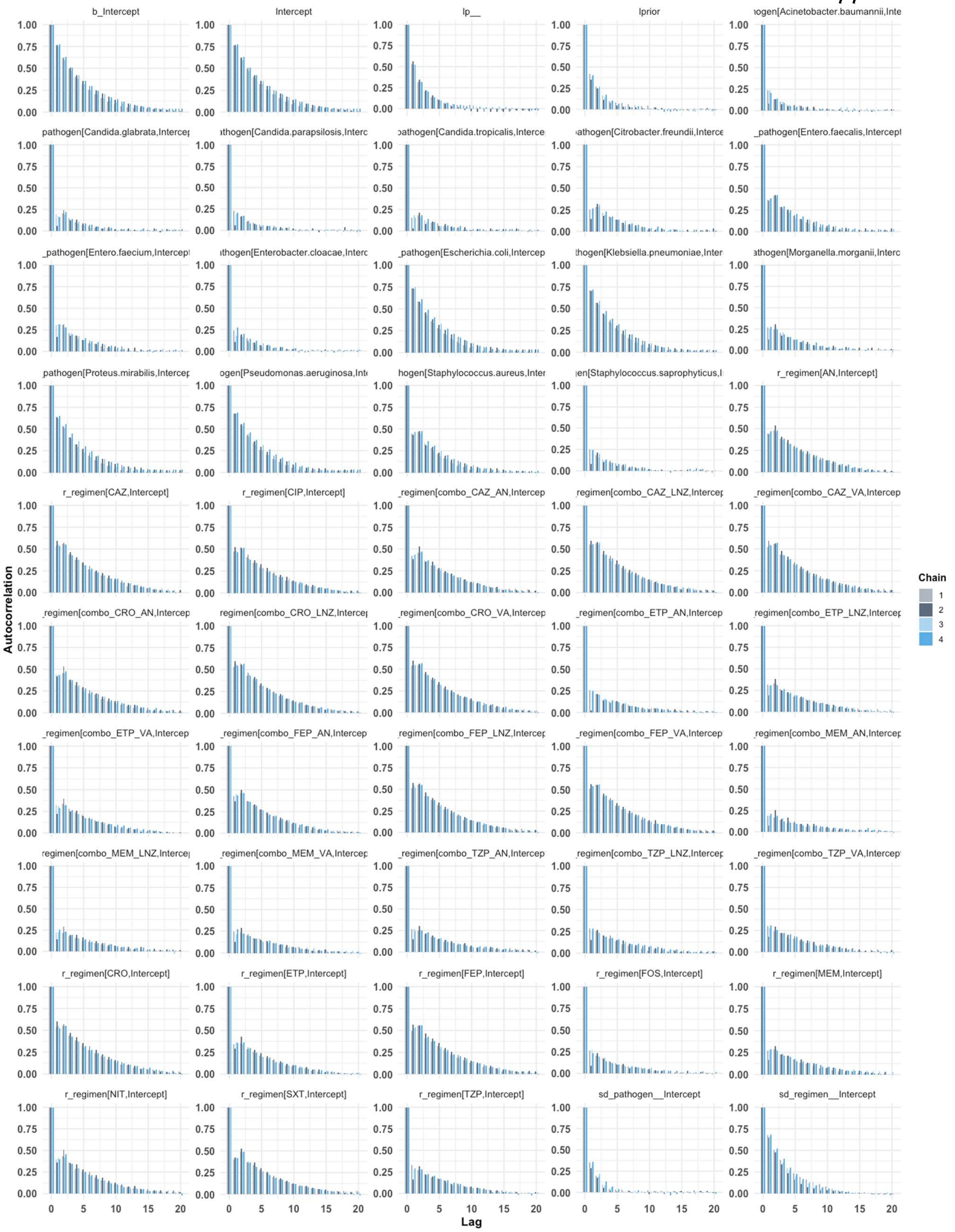
b_Intercept
Intercept
lp__
lprior
ıogen[Acinetobacter.baumannii,Inte
pathogen[Candida.glabrata,Intercep
ıthogen[Candida.parapsilosis,Interc
pathogen[Candida.tropicalis,Interce
athogen[Citrobacter.freundii,Interce
_pathogen[Entero.faecalis,Intercept
_pathogen[Entero.faecium,Intercept
ıthogen[Enterobacter.cloacae,Interc
_pathogen[Escherichia.coli,Intercep
thogen[Klebsiella.pneumoniae,Inter
athogen[Morganella.morganii,Interc
pathogen[Proteus.mirabilis,Intercep
ogen[Pseudomonas.aeruginosa,Int
hogen[Staphylococcus.aureus,Inter
ıen[Staphylococcus.saprophyticus,I
r_regimen[AN,Intercept]
r_regimen[CAZ,Intercept]
r_regimen[CIP,Intercept]
_regimen[combo_CAZ_AN,Intercep
regimen[combo_CAZ_LNZ,Intercep
_regimen[combo_CAZ_VA,Intercep
_regimen[combo_CRO_AN,Intercep
regimen[combo_CRO_LNZ,Intercep
_regimen[combo_CRO_VA,Intercep
_regimen[combo_ETP_AN,Intercep
regimen[combo_ETP_LNZ,Intercep
_regimen[combo_ETP_VA,Intercep
_regimen[combo_FEP_AN,Intercep
regimen[combo_FEP_LNZ,Intercep
_regimen[combo_FEP_VA,Intercep
_regimen[combo_MEM_AN,Intercep
regimen[combo_MEM_LNZ,Intercep
_regimen[combo_MEM_VA,Intercep
_regimen[combo_TZP_AN,Intercep
regimen[combo_TZP_LNZ,Intercep
_regimen[combo_TZP_VA,Intercep
r_regimen[CRO,Intercept]
r_regimen[ETP,Intercept]
r_regimen[FEP,Intercept]
r_regimen[FOS,Intercept]
r_regimen[MEM,Intercept]
r_regimen[NIT,Intercept]
r_regimen[SXT,Intercept]
r_regimen[TZP,Intercept]
sd_pathogen__Intercept
sd_regimen__Intercept
1.00
0.75
0.50
0.25
0.00
Autocorrelation
0
5
10
15
20
Lag
Chain
1
2
3
4
